# Heat Transfer Modulation of Micro-Textured Interfaces: A Multi-Scale Topology Optimization and Numerical Simulation

**DOI:** 10.3390/mi17060712

**Published:** 2026-06-10

**Authors:** Qing Rao, Benben Guo, Jiafu Ruan, Xigui Wang

**Affiliations:** 1 No. 6 Engineering (Xiamen), CCCC Third Harbor Engineering Co., Ltd., No. 189 Huachang Road, Huli District, Xiamen 361006, China; yaozhhit@126.com (Q.R.); chenxfhit@126.com (B.G.); 2 School of Mechatronics and Automation, Huaqiao University, No. 668 Jimei Avenue, Jimei District, Xiamen 361021, China; rjfnefu1993@126.com

**Keywords:** fish-attracting lamp, micro-textured interface, heat transfer modulation, multi-scale topology optimization, chimney-effect convection, heat pipe phase-change

## Abstract

To address the critical challenge of excessive junction temperature caused by ultra-high heat flux densities (>100 W/cm^2^) in deep-sea LED Fish-Attracting Lamp (FAL) arrays, this study proposes a hybrid thermal management scheme integrating interfacial micro-texturing, chimney-effect convection, and heat pipe phase-change heat transfer, achieving the unification of passive high-efficiency heat dissipation and pressure-resistant sealing. The FAL housing structure is reconfigured using topology optimization to construct chimney-effect enhanced flow channels integrated with heat pipe bundle arrays, thereby establishing efficient heat conduction pathways from the Phenolic Resin Substrate (PRS) to the structural periphery. Micro-Element Texture (MET) arrays are fabricated at the PRS thermal interface to enhance interfacial thermal conductance. Based on multi-physics coupled numerical simulation, a parametric mapping model correlating geometric topology with thermal performance is established through response interface methodology, enabling the parametric optimization of micro-texture configurations. A thermal interface performance testing platform is constructed to validate the accuracy and reliability of the numerical model. Experimental results demonstrate that the integrated heat pipe technology effectively suppresses LED junction temperature rise; moreover, groove-type MET arrays oriented perpendicular to the gravity direction not only significantly increase the effective heat dissipation area but also optimize the dynamic characteristics of natural convection. This proposed solution reduces the maximum operating temperature of deep-sea FALs by 6.70% compared with conventional structures, providing an effective engineering solution for thermal structural design of high-power illumination systems.

## 1. Introduction

As the core equipment of light-attracting fishing vessels, deep-sea fish aggregating FALs exploit the phototactic behavior of marine species to achieve fish schooling, representing a critical technical approach for enhancing fishing efficiency [1,2,3]. Currently, most fishing vessels still employ technically mature metal halide FALs as the light source. However, their kilowatt-scale power consumption and relatively low luminous efficacy result in substantial fuel costs that constrain the further improvement of economic benefits in deep-sea fisheries [4,5,6]. The advent of fourth-generation LED light sources has opened a new chapter in fish aggregating FALs technology: inherent advantages including extended service life, reduced power consumption, rapid response time, broad attraction range, excellent directional light emission, and superior underwater penetration capability collectively enable multiple benefits, maximized catch yield, decreased fuel consumption, and avoidance of ultraviolet damage, thereby significantly improving economic performance [7,8,9].

Nevertheless, with the continuous advancement of LED technology, deep-sea fish aggregating FALs are evolving toward higher power and brightness, imposing more stringent demands on thermal management system performance. During operation, high-power LED chips convert 70–80% of input electrical energy into heat. If this thermal energy cannot be dissipated promptly and effectively, elevated junction temperatures will occur, consequently triggering a cascade of issues including luminous efficiency degradation, accelerated material aging, and shortened operational lifespan [10,11,12]. Therefore, the design of efficient heat dissipation configurations is essential to ensure performance optimization and long-term reliable operation of high-power LED fish aggregating FALs.

Regarding natural convection cooling mechanisms, researchers have employed infrared thermography to conduct thermal characterization and comparative analysis of five distinct structural configurations of FALs [13,14]. The findings indicate that linear luminaires with non-chip-on-board (non-COB) packaging leverage their structural advantages to achieve effective thermal management through relatively simple passive cooling strategies. Conversely, COB-packaged luminaires necessitate high-performance active cooling systems to provide efficient heat dissipation pathways due to the concentrated heat flux densities generated during operation. In the domain of forced air cooling, investigators have developed integrated LED-FALs configurations combining fan-assisted cooling with passive heat dissipation [15,16,17]. The experimental apparatus utilized three continuous array arrangements with varying irradiation angles, aiming to optimize the installation spacing between fans and heat dissipation systems; however, the improvements in thermal performance remained limited.

Concerning liquid cooling technology exploration, prior studies have validated the application potential of multi-jet single-phase microchannel liquid cooling systems for thermal management of high-power LED lighting [18,19,20]. Distinguishing from the aforementioned conventional approaches, this study proposes an interface micro-texture enhanced heat dissipation configuration that synergistically integrates chimney effect with heat pipe phase-change heat transfer. By constructing functional micro-texture arrays on the external surface of the heat sink, the effective heat dissipation surface area is substantially expanded, and the gas–solid interface heat transfer mode is optimized, thereby significantly enhancing convective heat transfer efficiency. These research outcomes offer viable solutions and engineering references for addressing thermal management bottlenecks in deep-sea high-power LED-FALs systems.

Unlike existing studies that focus on single technological pathways such as heat pipe phase-change heat transfer, topology optimization of material distribution, or localized intensification by textured surfaces, this study addresses the compact cabin and high-humidity marine environment of LED fish-attracting lamps and proposes a composite heat dissipation architecture with the chimney effect as the core flow organization mechanism. This architecture drives global airflow through macro-scale buoyancy force, synergistically coupled with localized textured surface enhancement, and has formed distinctive technical characteristics from conventional thermal management solutions in terms of maintenance-free operation, attitude insensitivity, and structural compatibility.

## 2. Topology Optimization of Chimney-Effect-Enhanced Heat Dissipation Configurations

### 2.1. Topology Optimization Modeling for Heat Dissipation Configurations

In this study, thermal compliance is adopted as the objective function for the steady-state heat transfer problem to characterize the global nature of the optimization target. The minimization of this objective parameter aims to reduce the interface temperature of multi-scale configurations and enhance heat dissipation efficiency. The concept of thermal compliance is analogous to thermal potential energy. Minimizing thermal compliance enables maximized heat transfer to the surrounding medium, thereby achieving the optimal temperature distribution across the micro-textured interface [21,22,23]. Accordingly, the topology optimization of the heat dissipation interface configuration can be formulated as follows:

(1) $$\left\{\begin{array}{l} \mathrm{Find}\theta =\{\theta_{1},\theta_{2},\cdots ,x_{n}\}\in R^{n}\\ \min C={Τ}^{T}Κ(\theta )Τ=\sum_{e=1}^{n}{Τ}_{e}^{T}{Τ}_{e}\left(f_{e}(\theta_{e}){Κ}_{0}\right)\\ \sum_{e=1}^{n}\theta_{e}v_{e}\leq f\cdot V_{0}\\ 0<\theta_{\min}\leq \theta_{e}\leq 1\\ KT=R \end{array}\right.$$

where θ denotes the element density of the heat dissipation interface; C is the thermal dissipation deficiency, with units of (J⋅K); T is the temperature vector of the heat dissipation configuration interface nodes; K is the thermal conductivity matrix of the heat dissipation configuration interface; P is the thermal load vector of the heat dissipation configuration interface; ${Τ}_{e}$ is the temperature vector of the configuration element interface nodes; $\theta_{e}$ is the relative density of each material element; $v_{e}$ is the interface element volume; $V_{0}$ is the total volume of the heat dissipation interface configuration design domain; f is the material volume fraction; n is the total number of interface elements; $\theta_{\min}$ is the minimum interface element density to prevent singular matrices during computation, typically set to $1.0\times 10^{-3}$; Κ indicates the interfacial element thermal conductivity matrix at unit relative density (ρ=1.0) within the heat dissipation topology design domain; $f_{e}(\theta_{e})$ is an artificially defined exponential function model, which can be expressed as $f_{e}(\theta_{e})=\theta_{e}^{P}$.

**Figure 1:**
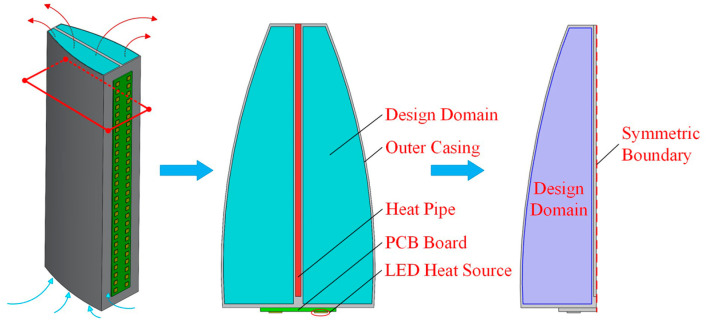
Model construction for topology optimization of heat dissipation configurations using equivalent simplification strategies.

Figure 1 presents the heat dissipation structure model for micro-textured interfaces utilizing the chimney effect. The blue region denotes the topology optimization design domain, while the remaining areas retain their original configurations. Considering that full three-dimensional topology optimization of chimney-configured micro-textured interface heat dissipation structures tends to yield geometrically irregular results with prohibitive computational costs, this study employs an equivalent simplification strategy: the three-dimensional model is subjected to cross-sectional slicing, numerical simulations are conducted on the simplified two-dimensional model, and the optimized results are subsequently extruded to reconstruct the three-dimensional chimney configuration, ultimately forming a heat dissipation structure with micro-textured interfaces.

### 2.2. Thermal Dissipation Deficiency Analysis Considering the Chimney Effect

The thermal design of the deep-sea FALs’ heat dissipation configuration is based on the chimney effect enhancement principle. By optimizing the natural convection channels within the heat dissipation structure, the aerodynamic driving efficiency is enhanced, thereby improving the natural convection heat transfer coefficient in the structural domain of the FALs and achieving a systematic improvement in overall heat dissipation performance [24,25,26]. This configuration converts the buoyancy force generated by density differences arising from temperature gradients between the internal and external regions into the dominant driving force for airflow motion [27,28,29]. The thermodynamic behavior can be quantitatively described by the following governing equations [30,31]:

(2) $$\Delta P=(\rho_{c}-\rho_{h})gH$$

where ΔP is the buoyancy pressure difference (Pa), $\rho_{c}$ is the density of cold air, $\rho_{h}$ is the density of hot air, $\rho_{c}=\rho_{h}=101,325/287\cdot T$ ($\mathrm{kg}/m^{3}$), herein, T is the absolute temperature (K), g is the gravitational acceleration ($m/s^{2}$), and H is the vertical height of the micro-textured heat dissipation shell (chimney-shaped) in millimeters (mm).

In chimney-driven natural convection, buoyancy-induced flow is characterized by the Rayleigh number (Ra), which determines the resulting flow regime [32,33].

(3) $$Ra=G_{r}\cdot P_{r}$$

where $G_{r}$ denotes the Grashof number, $G_{r}=\beta g\Delta TH^{3}/\upsilon^{2}$, a larger Grashof number indicates stronger buoyancy effects in the fluid. $P_{r}$ represents the Prandtl number, $P_{r}=\upsilon /\alpha$, υ is the kinematic viscosity of air, $\upsilon \approx 1.8\times 10^{-5}\;m^{2}/s$, and α is the thermal diffusivity ($m^{2}/s$). For air, $P_{r}\approx 0.7$. β denotes the thermal expansion coefficient, for an ideal gas, $\beta ={1/T}_{h}$($K^{-1}$).

The internal air velocity is determined by the interplay between buoyancy and flow resistance [34,35]. Under the assumption of steady laminar flow within simplified configurations, the air velocity can be derived as follows [36]:

(4) $$v=\sqrt{\frac{2\Delta P}{\rho_{h}\left(K_{i}+K_{o}+\lambda \frac{H}{d_{h}}\right)}}$$

where the local resistance coefficients for inlet ($K_{i}$) and outlet ($K_{o}$) are defined as follows: For a sharp-edged inlet, $K_{i}\approx 0.5$; if the inlet is rounded, then $K_{i}\approx 0.04\sim 0.2$. Since the outlet discharges directly to atmosphere, $K_{i}\approx 1.0$. λ represents the channel friction coefficient, $d_{h}$ denotes the chimney structural channel diameter (mm). For irregular geometric cross-sections, the hydraulic diameter $d_{h}=4A/C$ can be employed (where A is the cross-sectional area and C is the wetted perimeter).

### 2.3. Thermal Simulation Configuration and Boundary Conditions

**Figure 2:**
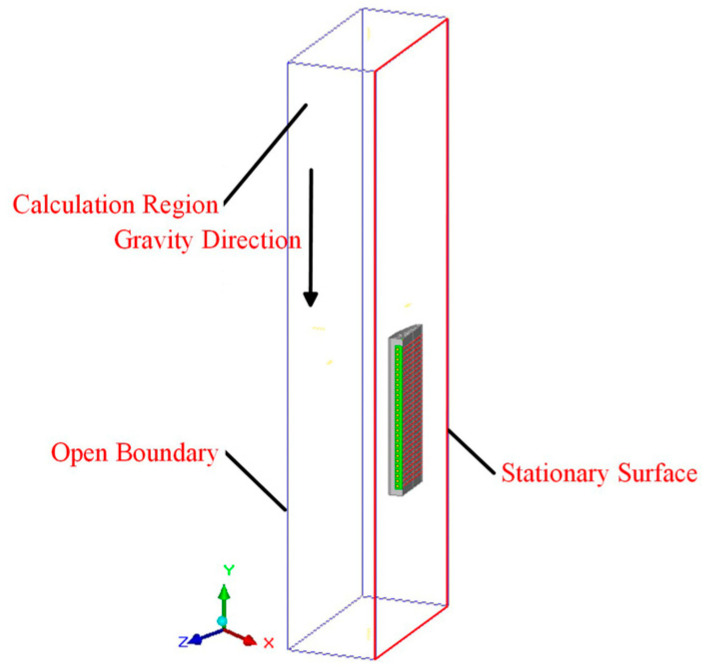
Computational domain of the simulation model.

To reduce computational resource requirements, the LED chip and Phenolic Resin Substrate (PRS) are geometrically simplified, and the thermal contact resistance between interfaces is neglected in the heat dissipation analysis. Thermal simulations are performed using Ansys 2023 R2 Icepak software. Owing to the geometric complexity, only half of the model is simulated. The symmetry plane is defined as an adiabatic wall with zero heat flux, while all other surfaces are treated as open boundaries to permit natural convection. The temperature boundary condition at the maximum Y-coordinate is removed, and the simulation model is illustrated in Figure 2. The materials employed in this study are as follows: 6063 aluminum alloy is used for fabricating the heat dissipation structure; a high-thermal-conductivity metal substrate is adopted for the PRS board; and silicon serves as the material for the LED chip. Heat pipes are incorporated for subsequent thermal simulations, with the relevant material properties detailed in Table 1. Each LED chip is designated as a 7.0 W heat source, corresponding to 70% of its total thermal power (assuming a typical electro-optical conversion efficiency of 30%).

The Discrete Ordinates (DO) radiation model, which is suitable for open systems with natural convection, is employed in this study. The Rayleigh number (Ra) is calculated using Equation (3) as $Ra=2.878\times 10^{8}<10^{9}$, indicating that the airflow under natural convection is laminar. The gravitational force acts in the −Y direction. Additionally, considering the high ambient temperatures encountered during marine operations of the FAL, the ambient temperature is set to 30 °C.

### 2.4. Geometric Configuration Design of the Capillary Suction Tube

**Figure 3:**
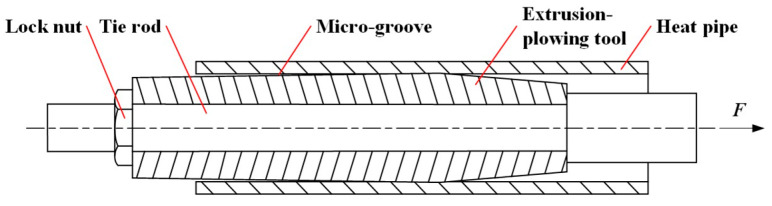
Principles of micro-groove plowing on the inner surface of heat pipes.

**Figure 4:**
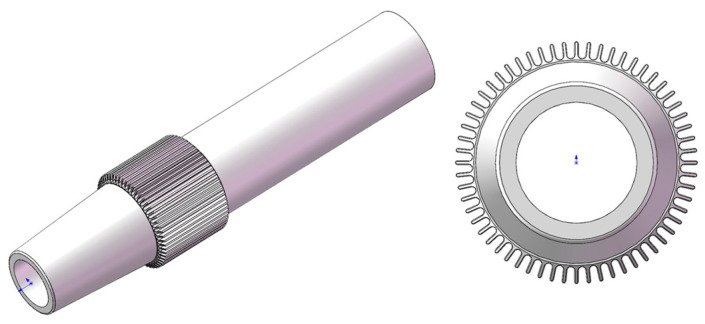
Schematic diagram of the extrusion-plowing tool.

Considering both the machining constraints of the monolithic heat pipe and the operating environment of the fish-attracting light, a grooved heat pipe is selected as the capillary wicking core structure. Leveraging the excellent plastic deformation capability of aluminum alloy 6063, a metal plowing process is adopted to fabricate the microgrooves in the monolithic heat pipe, with the forming principle illustrated in Figure 3. Prior to the extrusion-plowing operation, the heat sink structure of the FAL is firmly secured in position, while the extrusion-plowing tool is mounted onto the pull rod via a locking nut and precisely aligned with the heat pipe opening. Upon initiation of the extrusion-plowing process, the pull rod advances slowly along the inner wall of the heat pipe under external force; the extrusion-plowing tool then exerts compressive stress on the inner wall, inducing plastic deformation and lateral material flow. As the metal continuously bulges outward at both ends, a capillary wick structure with continuous fins is formed within the microgrooves of the heat pipe. A schematic diagram of the extrusion-plowing tool is presented in Figure 4.

**Figure 5:**
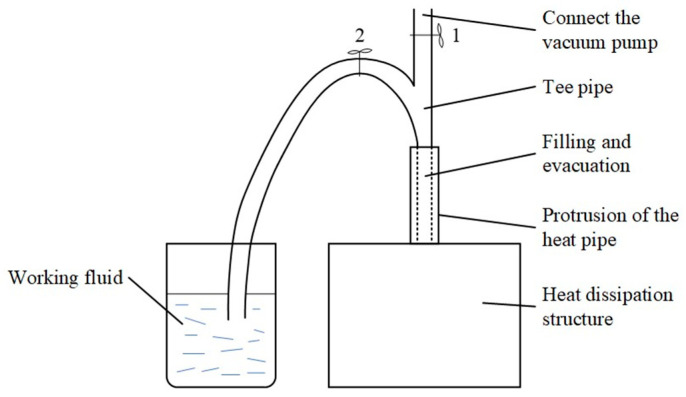
Schematic diagram of the working fluid charging and vacuum evacuation process.

As the tube shell of the integrated heat pipe serves as the heat dissipation structure, acetone, compatible with 6063 aluminum alloy, is selected as the working fluid. Figure 5 illustrates the procedures and vacuum evacuation of the heat pipe. To facilitate fluid charging, vacuum evacuation, and subsequent sealing during the fabrication of the heat dissipation structure, an extension tube is incorporated at the reserved heat pipe location.

Acetone is employed as the working medium for the integrated heat pipe, considering that the tube shell functions as the primary heat dissipation component, and acetone exhibits favorable chemical compatibility with 6063 aluminum alloy. Figure 5 depicts the sequential procedures for working fluid charging and vacuum evacuation. To accommodate the requirements of fluid charging, vacuum evacuation, and ultimate hermetic sealing during the fabrication process, an extension tube is integrated at the designated heat pipe port. The T-joint is hermetically affixed to the protruding extension tube via sealing material. During vacuum evacuation, Channel 2 is occluded, permitting the vacuum pump to degas the heat pipe interior through Channel 1. For fluid charging, Channel 1 is sealed, and the working fluid is introduced to the specified fill volume by exploiting the pressure gradient between the evacuated cavity and ambient atmosphere. In the final sealing operation, both channels are sealed, and the protruding extension tube is mechanically crimped and truncated using a high-temperature sealing apparatus to establish permanent hermetic integrity.

### 2.5. Multi-Scale Micro-Element Configuration Design for Textured Surfaces

To investigate the thermal dissipation performance of heat sink structures in fish-attracting lights under various surface textures, five distinct surface texture configurations are designed in this study, all featuring a uniform depth of 0.2 mm, as detailed in Table 2.

**Figure 6:**
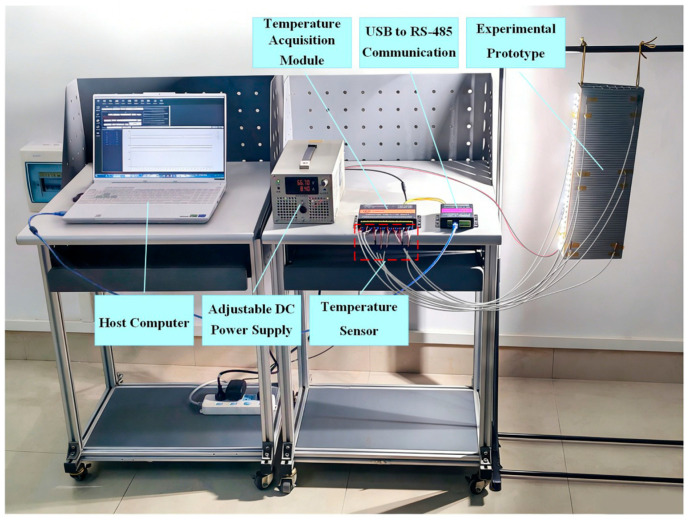
Experimental platform for thermal management testing FALs.

To validate the simulation results, a heat dissipation test platform for the FAL is developed, as shown in Figure 6. The experimental setup comprises a test prototype, an 1800 W adjustable DC power supply, a temperature acquisition module, temperature sensors, and other auxiliary components.

## 3. Topology Optimization and Simulation Analysis

**Figure 7:**
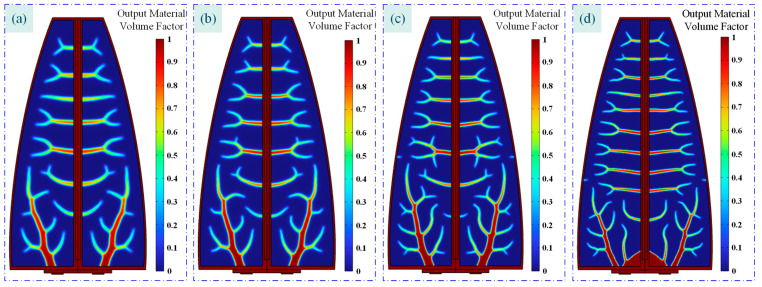
Topology optimization of LED substrates for deep-sea FALs under varying filter radii. (**a**) $R_{\min}=3.0\;MES$, (**b**) $R_{\min}=2.5\;MES$. (**c**) $R_{\min}=2.0\;MES$. (**d**) $R_{\min}=1.5\;MES$.

Considering the 0.6 mm thickness of the LED substrate for deep-sea FALs, the maximum Mesh Element Size (MES) is set to 0.6 mm. With the mesh held constant, the influence of varying filter radii ($R_{\min}$) on the topology optimization results of the substrate structure is systematically investigated, as shown in Figure 7.

**Figure 8:**
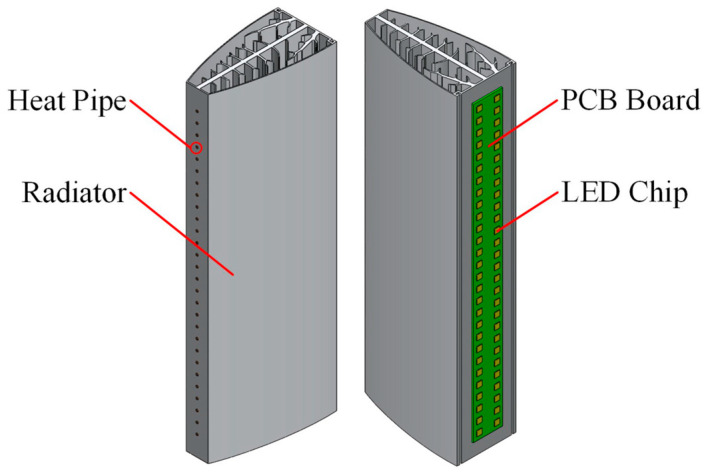
Three-dimensional solid model of the deep-sea FAL.

Figure 7a reveals that an excessively large filter radius leads to over-smoothing of the deep-sea FAL substrate topology, accompanied by the loss of fine-scale features and a consequent degradation in structural service performance. Conversely, an overly small filter radius (as illustrated in Figure 7c,d) generates jagged boundaries and proliferates numerous microscopic branches. Although these microstructural features are conducive to enhancing interfacial heat transfer efficiency, their dimensions transcend the manufacturability limits of conventional machining, casting, and additive manufacturing processes. Moreover, they are prone to fracture failure under vibratory or thermal loading. Therefore, an optimal filter radius must be determined through a comprehensive trade-off between topological fidelity and manufacturability. Based on the optimized topology, a three-dimensional solid model of the deep-sea FAL is established, as shown in Figure 8.

### 3.1. Comparative Simulation Analysis of Surface Texture Micro-Element Configurations for the Proposed FAL Model

Table 3 presents the comparative simulation results for various surface textures. Among the proposed configurations, grooves oriented perpendicular to the gravity direction yield the most significant improvement in the heat dissipation performance of the FAL system. These grooves function as a series of miniature dams along the upward path of natural convection airflow, periodically disrupting the thermal boundary layer and enhancing fluid mixing, thereby promoting more effective heat exchange between the near-surface air and the heat dissipation structure. In contrast, grooves aligned parallel to the gravity direction act in concert with the primary flow, guiding air upward along the channels; this behavior tends to stabilize or even thicken the thermal boundary layer, resulting in degraded heat transfer in certain regions. While dimple-type textures, including circular and rectangular pits, can also disturb the thermal boundary layer to enhance local heat transfer, their effects remain localized and discrete. As airflow passes over these dimples, stable recirculating vortices develop within the cavities; the poor exchange between the recirculation zone and the mainstream weakens convective heat transfer, rendering the overall thermal performance of the FAL inferior to that achieved with perpendicular grooves, which enable continuous and complete disruption of the boundary layer. Accordingly, this study adopts grooves perpendicular to the gravity direction as the optimal micro-scale surface texture configuration.

### 3.2. Optimization Analysis of Multi-Scale Configuration Parameters for Surface Micro-Textures

This study employs the response surface methodology, with the multi-scale configuration parameters of surface micro-texture, namely, groove width (*W*), groove spacing (*S*), and groove depth (*D*), as independent variables, to establish a response relationship model that correlates these parameters with the maximum temperature of the deep-sea FAL structure. These parameters are further treated as associated functions of the multi-scale configuration parameters of surface micro-texture, thereby elucidating the time-varying characteristics of this temperature extremum. Table 4 lists the experimental factors, along with the corresponding coded levels employed in the response surface design.

The simulation analysis results obtained using Ansys Icepak (2024 R2), together with the corresponding simulation validation test protocols, are summarized in Table 5. Statistical analysis of the simulation validation test data is performed using Design-Expert 13 (v13.x) to evaluate the effects of individual experimental factors on the response variables.

Based on the experimental data presented in Table 5, a second-order response surface regression model is developed using response surface methodology, with groove texture parameters as independent variables and interface temperature as the response variable.

(5) $$\begin{array}{c} T=110.49297-0.087188W-0.129364S-2.90211D-0.000795WS-0.071595WD\\ \;\;\;\;-0.006187SD+0.021239W^{2}+0.019739S^{2}+7.38636D^{2} \end{array}$$

**Figure 9:**
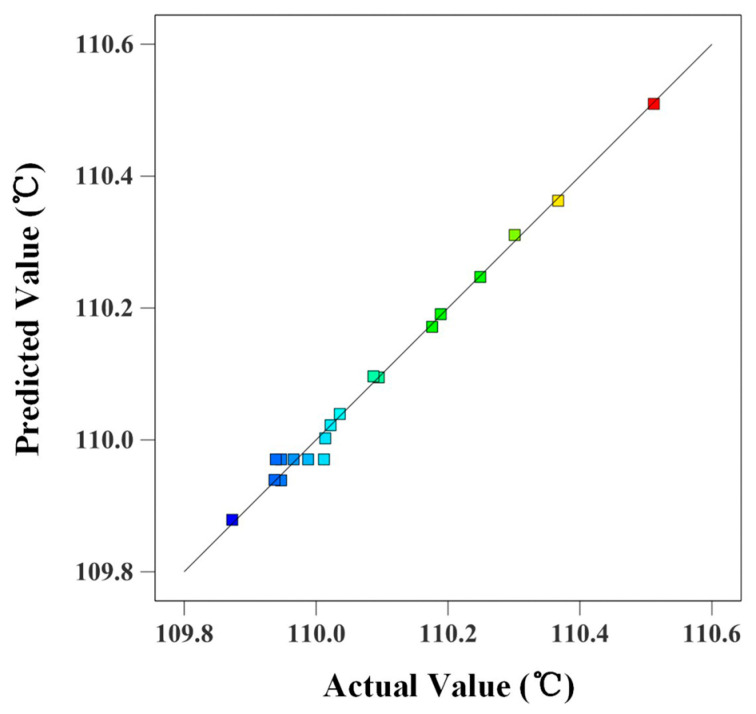
Comparison of predicted and actual maximum temperatures.

A second-order response surface methodology is employed, with groove-type micro-texture parameters serving as input variables, to establish a regression-based prediction model for the maximum interface temperature. The model exhibits excellent agreement with simulation data, and the corresponding temperature evolution is presented in Figure 9.

**Figure 10:**
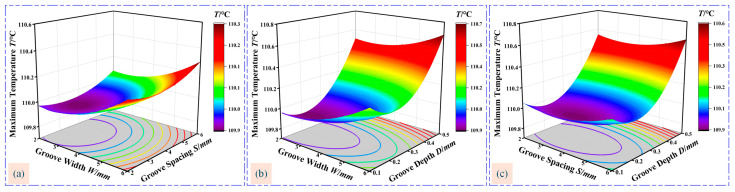
Time-varying coupling characteristics between multiscale parameters of groove-type micro-texture configurations and the maximum temperature response surface of FAL structural interfaces. (**a**) Variation in *W* and *S*, (**b**) variation in *W* and *D*, (**c**) variation in *S* and *D*.

To further elucidate the coupled relationship between the multi-scale parameters of grooved micro-texture configurations and the peak interfacial temperature of FAL structures, a time-dependent model is developed using response surface methodology, with the results presented in Figure 10. Figure 10a–c illustrate the interactive effects of pairwise micro-texture parameter combinations on the maximum interface temperature. Through quantitative resolution of the coupling intensity among multi-scale parameters, a comprehensive evaluation of the overall heat dissipation performance of the deep-sea FAL is achieved.

The contour density variations indicate that the multi-scale parameters of the groove-type micro-texture configuration affect the maximum interface temperature with the following order of significance: groove depth (D) > groove width (W) > groove spacing (S). Among these, groove depth (D) exerts the most pronounced influence on the thermal performance of the FAL structure, and its underlying mechanisms can be elucidated from the following two aspects. (1) Convective enhancement effect. The groove depth directly governs the disturbance intensity of the thermal boundary layer and the vortex generation capability. As the groove depth increases, the thermal boundary layer undergoes more severe disturbances, thereby inducing stronger vortices and enhancing convective heat transfer. Nevertheless, this convective improvement is not monotonic. Once the groove depth exceeds a critical value, the benefits derived from thermal boundary layer disturbances diminish markedly, while the flow resistance coefficient rises sharply, causing the overall effectiveness of convective enhancement to decline instead. (2) Thermal conduction weakening effect. The attenuation of solid-phase heat transfer paths by groove depth exhibits spatial non-uniformity. The core function of the heat sink is to rapidly conduct the heat generated by the LED chip to the FAL substrate; however, as the groove depth increases, the wall thickness at the groove root progressively decreases, gradually obstructing the heat conduction path from the heat sink toward the region away from the LED chip. An optimal balance must be sought between the convective enhancement benefits brought by increased groove depth and the concomitant weakening of the heat conduction paths, so as to maximize the overall thermal performance of the FAL.

Groove width (W) is a secondary factor influencing the maximum interfacial temperature of the FAL structure. When W is too small, the grooves form narrow micro-slits. At the groove entrance, the airflow experiences severe contraction, preventing most of the air from penetrating into the groove interior; only weak turbulent eddies develop along the groove edges, resulting in inefficient convective heat transfer. Conversely, an excessively large W is equivalent to the continuous removal of a segment of the FAL substrate, reducing the effective heat conduction cross-sectional area and significantly impeding thermal conduction.

Groove spacing (S) is the least significant factor affecting the maximum interfacial temperature of the FAL structure. During surface texturing, material removal to form grooves results in a wall thickness at the groove spacing that exceeds that at the groove width (the difference being the groove depth D). Thus, the groove spacing effectively corresponds to the width of the heat dissipation fins. An excessively small spacing increases the fin count but makes each individual fin thinner, degrading heat transfer capability and preventing heat from being effectively transferred from the proximal end adjacent to the LED heat sink to the distal end. Conversely, an excessively large spacing produces an insufficient number of grooves to effectively disrupt the thermal boundary layer.

Based on Equation (5), a mathematical model is formulated with the maximum interfacial temperature as the optimization objective, aiming at the global optimization of the overall thermal performance of the FAL structure. The objective function is defined in terms of groove parameters, which yields the following expression:

(6) f(W,S,D)=T

Throughout the parameterized multi-scale design of micro-texture configurations, the determination of groove geometric parameters is restricted by multiple coupled practical engineering limitations, thereby ensuring the optimized configurations are readily manufacturable in engineering practice.

(7) $$\left\{\begin{array}{l} 0.5\;\mathrm{mm}\leq W\leq 10\;\mathrm{mm},\;W\in R\\ 0.5\;\mathrm{mm}\leq S\leq 10\;\mathrm{mm},\;S\in R\\ 0.05\;\mathrm{mm}\leq D\leq 0.8\;\mathrm{mm},\;D\in R \end{array}\right.$$

A genetic algorithm is implemented on the MATLAB R2025a platform to perform multi-objective optimization of multi-scale parameters for groove-type micro-textures in the FAL structure. Following iterative refinement, the optimal parameter combination is identified as: groove width W = 2.48 mm, spacing S = 3.37 mm, and depth D = 0.22 mm. With this optimal configuration, the peak interfacial temperature of the FAL structure is reduced to 109.868 °C, thereby effectively enhancing the thermal management performance of the deep-sea FAL.

### 3.3. Comparison and Mechanistic Analysis of Thermal Simulation Results

Table 6 presents a comparative analysis of simulation results for three multi-scale micro-textured structural configurations: (1) without heat pipes and without surface textures; (2) with heat pipes but without surface textures; and (3) a synergistic integration of heat pipes with groove micro-textures oriented perpendicular to the gravity direction (based on the aforementioned optimal parameters).

The interface temperature contours presented in Table 6 reveal that integrating heat pipes significantly homogenizes the temperature distribution across the FAL heat dissipation structure. This improved temperature uniformity effectively reduces the thermal gradient between the proximal and distal ends of the heat pipe, thereby mitigating heat accumulation in the PRS region. The optimized interface temperature field enhances overall convective heat transfer performance, effectively lowering the LED junction temperature. With the further incorporation of surface textures, the maximum interface temperature of the FAL exhibits an additional reduction compared to the configuration integrating heat pipes alone (without texturing). This enhancement is primarily attributed to the extended heat dissipation area and intensified airflow turbulence induced by surface textures, which synergistically augment the thermal performance of the FAL. These results collectively demonstrate that both heat pipes and surface textures exert independent yet significant effects in reducing the peak interface temperature of the FAL. Specifically, the optimal design employing heat pipes coupled with gravity-assisted vertical grooves (at optimal multi-scale parameters) achieves a maximum temperature reduction of 6.73% relative to the baseline luminaire without heat pipes or textures. Furthermore, the deviation between the simulation results of this optimal design and the predictions of the second-order response surface regression model is merely 0.021 °C, fully validating the reliability of the optimization model.

The global airflow velocity slice contours (see Table 6) indicate that air velocity increases monotonically with chimney height, displaying a pronounced bottom-to-top velocity gradient. This suggests that the chimney effect serves as the dominant driving force for system heat dissipation. Although the synergistic integration of heat pipes and surface textures renders the airflow velocity distribution more uniform, the overall flow velocity decreases slightly. This phenomenon can be explained as follows: the temperature difference between the chimney outlet and inlet constitutes the core driving force for internal airflow motion; as this temperature difference gradually diminishes, the chimney effect weakens correspondingly, leading to a reduction in airflow velocity.

## 4. Comparison of Simulation and Experimental Results

**Figure 11:**
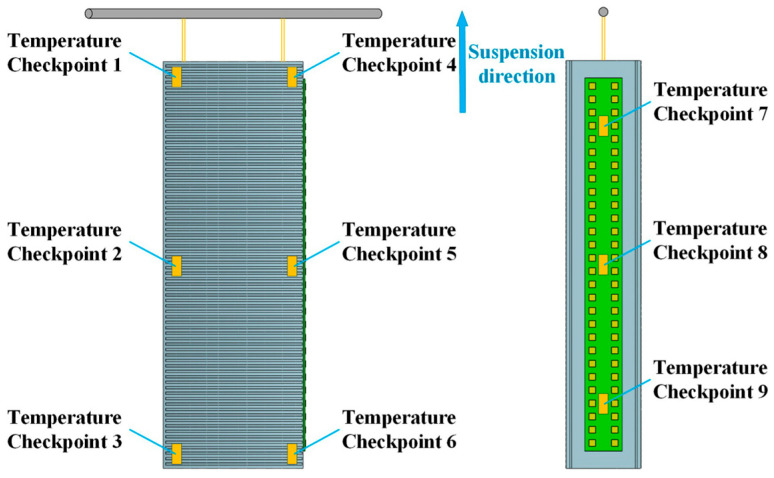
Temperature measurement point location at the deep-sea FAL interface.

To enable accurate monitoring of the interfacial temperature distribution, six temperature measurement points are deployed on the FAL heat dissipation structure, as illustrated in Figure 11, supplemented by three additional monitoring points on the PRS surface. The experiments are performed in a sealed environment maintained at a constant temperature of 30 °C, with temperatures at all measurement points being continuously recorded in real time until the FAL system attained thermal steady-state operation.

**Figure 12:**
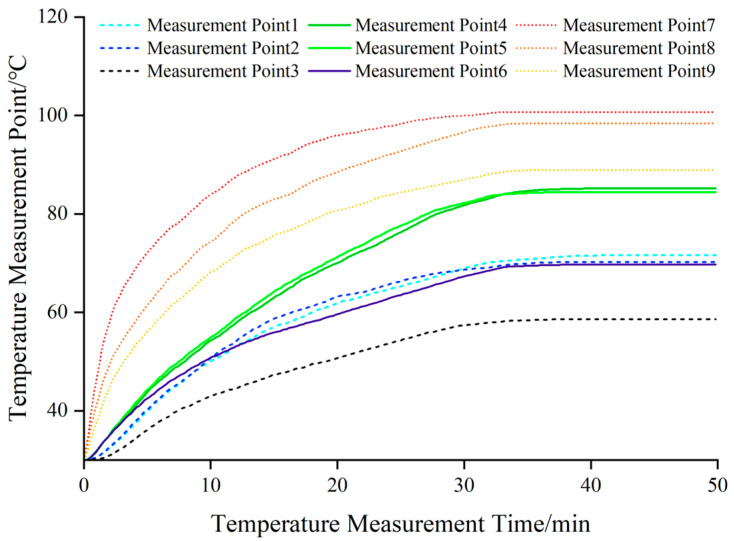
Temporal evolution of the FAL interfacial temperature rise at the measurement location.

To further ensure the reliability of the experimental results, three independent replicates are performed under identical operating conditions. The interface temperature acquisition module is configured to automatically record and store data at 10 s intervals, with a single experimental duration of 50 min. This duration is selected to ensure that the FAL system fully reaches thermal equilibrium and the temperature rise stabilizes. Given that the temperature–time curves obtained from the three experiments exhibited highly consistent trends and the numerical deviations at corresponding measurement points are minimal, the temperature data at the same time instant and measurement location across the three experiments are averaged arithmetically. This approach simplifies data presentation while accurately reflecting the average thermal performance of the test object, yielding the temperature-rise curves for each measurement point as shown in Figure 12.

**Figure 13:**
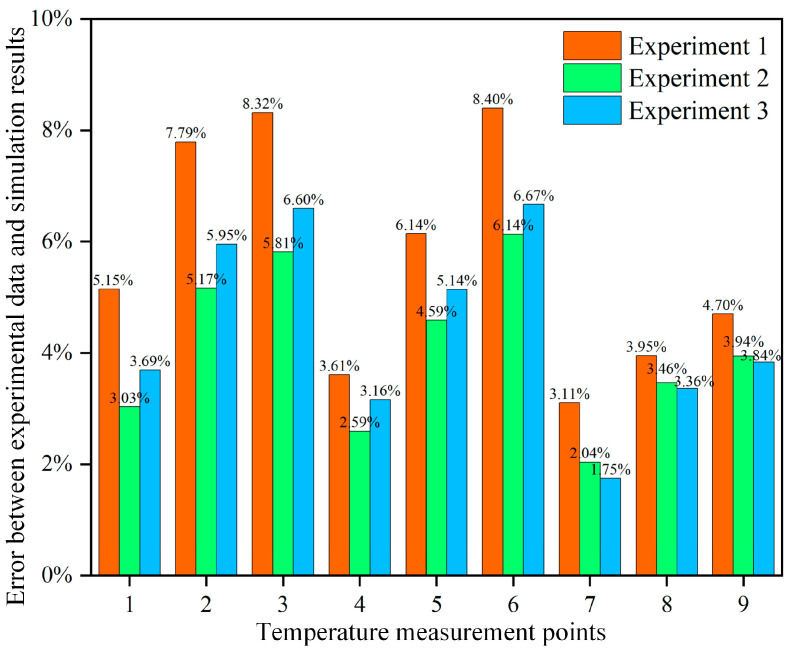
Temporal evolution of discrepancy curves between experimental and numerical simulation errors in deep-sea friction-and-lubrication interface measurements.

In Figure 13, the experimental data and simulation results for the nine interface temperature monitoring points are compared and analyzed. Although a certain temperature deviation exists between the experiments and simulations, the relative deviation for all measurement points is within 10%, indicating good agreement between the two. This level of consistency confirms that the simulation model possesses sufficient computational accuracy and reliability, providing a sound theoretical basis and engineering guidance for the thermal management design of the FAL system.

**Figure 14:**
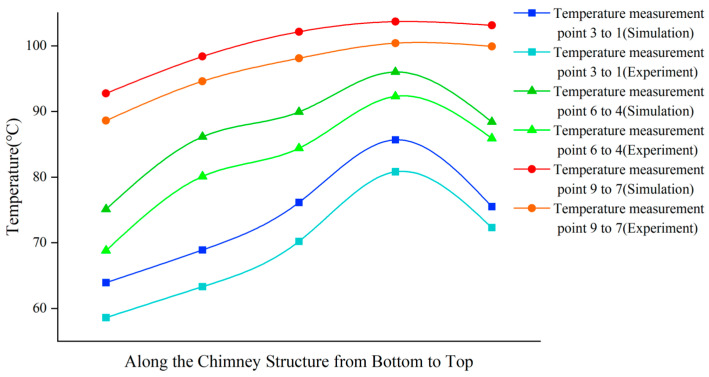
Comparison of experimental and simulated interfacial temperature variations along the chimney-shaped FAL structure from bottom to top.

To characterize the temperature distribution along the chimney structure, intermediate monitoring points are added between measurement points 3 and 2, and between points 2 and 1, on the heat dissipation structure away from the PRS side. These points are intended to capture the gradual temperature variation from the chimney bottom to the top. On the side close to the PRS and across the PRS interface region, midpoint sensors are correspondingly installed between points 6 and 5, 5 and 4, 9 and 8, and 8 and 7, to finely resolve the temperature field distribution characteristics on that side and at the interface. The comparison between experimental measurements and numerical simulations is shown in Figure 14. Although there are some deviations in temperature values at individual measurement points, the overall trend of the FAL temperature rise curve agrees well, thereby validating the reliability of the numerical model.

## 5. Conclusions

Heat pipes and surface texturing represent two critical technological pathways for enhancing the thermal performance of micro-element configurations in deep-sea FAL systems. By evaluating highly efficient phase-change heat transfer mechanisms, heat pipes effectively suppress heat accumulation near the heat source, rapidly transporting heat generated by LED chips from the proximal to the distal end of the heat sink, thereby achieving spatial redistribution and complete dissipation of thermal energy. Surface texturing, conversely, augments the effective heat dissipation area and induces turbulent airflow, synergistically intensifying convective heat transfer.

This study systematically evaluates the thermal characteristics of three micro-textured interface configurations through numerical simulation: a baseline configuration without heat pipes or texturing, an improved configuration integrating heat pipes without texturing, and an optimized configuration simultaneously coupling heat pipes with vertically oriented gravity-direction grooves. Simulation results demonstrate that heat pipes significantly reduce the temperature gradient between the proximal and distal regions of the heat sink. By alleviating localized thermal accumulation around the heat source, heat pipes enhance the overall convective heat transfer intensity of the FAL system and effectively reduce the junction temperature of LED modules. Furthermore, machining vertical grooves on the heat sink surface periodically disturbs the thermal boundary layer and enhances fluid mixing, thereby promoting more thorough heat exchange between the solid surface and the surrounding air.

The thermal performance of the synergistic optimization design combining heat pipes and vertical surface grooves is further validated through experimental measurements, with results compared against numerical predictions. Although minor deviations exist between experimental temperature data and simulated values, both exhibit excellent consistency in their variation trends, with all relative errors controlled within 10%.

## Figures and Tables

**Table 1:** The relevant material properties.

Configuration Name	Thermal Conductivity (W/(m×K))	Density ($\mathrm{kg}/m^{3}$)
Heat dissipation structure	201	2700
PRS board	201 (XY direction)	3000
	6 (Z direction)	
LED chip	149	2330
Heat pipe	201 (XY direction)	2000
	10,000 (Z direction)	

**Table 2:** Micro-element texture configuration design for heat dissipation structures of FALs.

Textural Type	Schematic Diagram	Parameter Diagram
Groove (parallel to the direction of gravity)	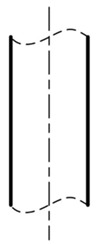	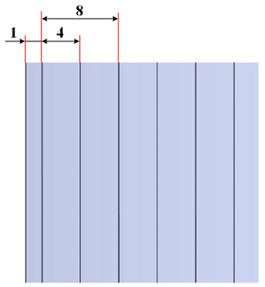
Groove (perpendicular to the direction of gravity)	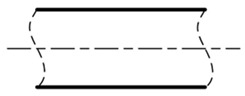	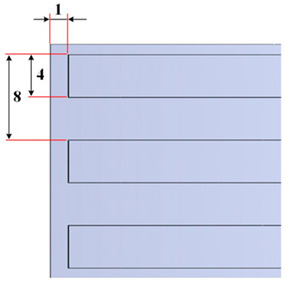
Hexagonal indentation	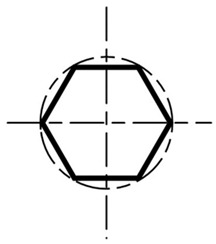	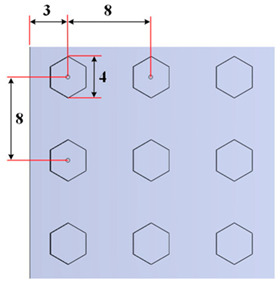
Rectangular indentation	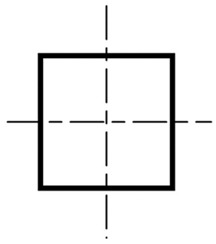	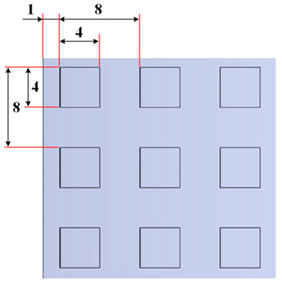
Circular indentation	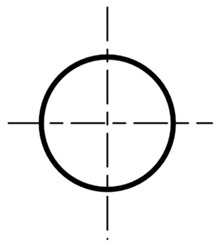	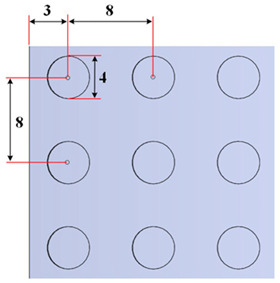

**Table 3:** Comparative simulation of temperature contours for different micro-scale surface texture configurations.

Textural Type	X-Direction Temperature Contour Map	Textural Type	X-Direction Temperature Contour Map
Groove (parallel to the direction of gravity)	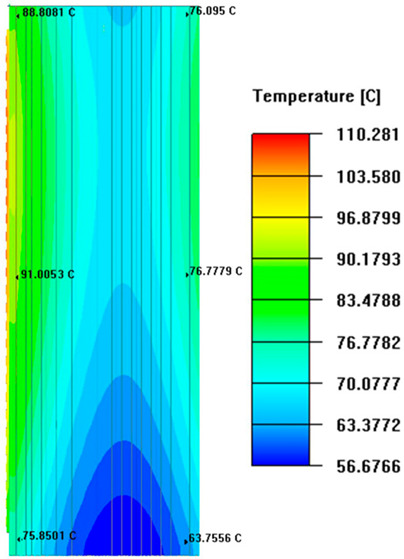	Groove (perpendicular to the direction of gravity)	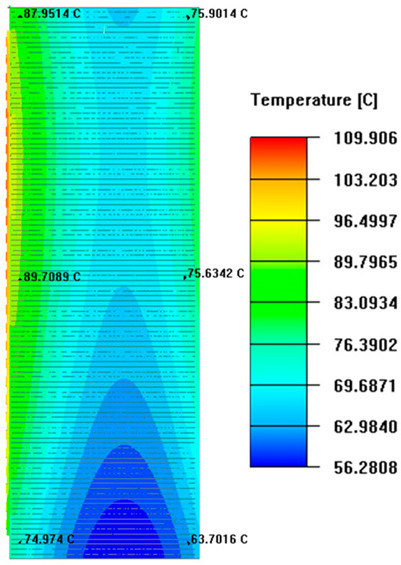
Hexagonal indentation	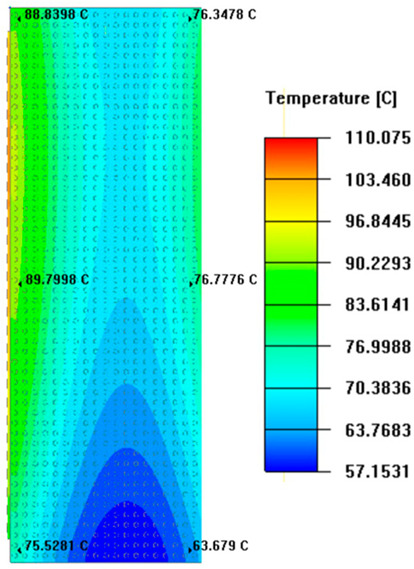	Rectangular indentation	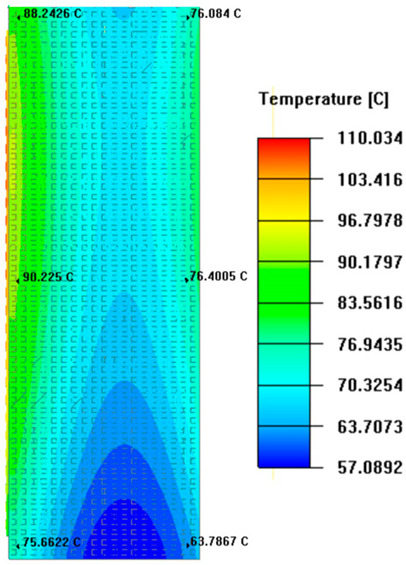
Circular indentation	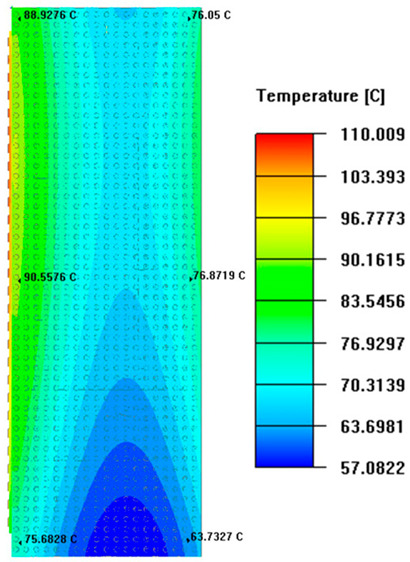	No texture	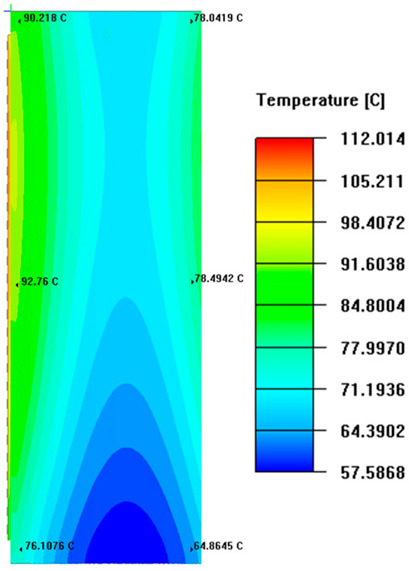

**Table 4:** The experimental factors and the coded levels.

Level	W (mm)	S (mm)	D (mm)
1.682	6.00	6.00	0.50
1.000	5.19	5.19	0.42
0.000	4.00	4.00	0.30
−1.000	2.81	2.81	0.18
−1.682	2.00	2.00	0.10

**Table 5:** Correspondence between simulation results and validation test schemes.

Group	Experimental Factors			Response Value
	W (mm)	S (mm)	D (mm)	T (°C)
1	−1.00 (2.81)	−1.00 (2.81)	−1.00 (0.18)	109.873
2	1.00 (5.19)	−1.00	−1.00	110.036
3	−1.00	1.00 (5.19)	−1.00	109.947
4	1.00	1.00	−1.00	110.095
5	−1.00	−1.00	1 (0.42)	110.189
6	1.00	−1.00	1.00	110.301
7	−1.00	1.00	1.00	110.249
8	1.00	1.00	1.00	110.367
9	−1.683 (2)	0.00 (4)	0.00 (0.30)	109.937
10	1.683 (6)	0.00	0.00	110.176
11	0.00 (4)	−1.683 (2)	0.00	110.014
12	0.00	1.683 (6)	0.00	110.087
13	0.00	0.00	−1.683 (0.10)	110.022
14	0.00	0.00	1.683 (0.50)	110.512
15	0.00	0.00	0.00	109.966
16	0.00	0.00	0.00	109.947
17	0.00	0.00	0.00	109.939
18	0.00	0.00	0.00	109.988
19	0.00	0.00	0.00	110.012

**Table 6:** Comparative analysis of FAL simulation results for different multi-scale micro-texture configurations.

	No Heat Pipes and No Surface Texturing	With Heat Pipes but Lacking Surface Texturing	Incorporating Both Heat Pipes and Grooves Oriented Perpendicular to the Direction of Gravity
Isometric temperature cloud map	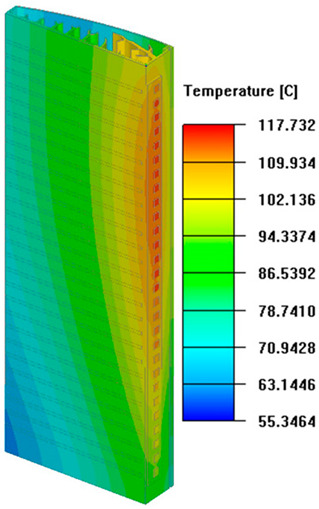	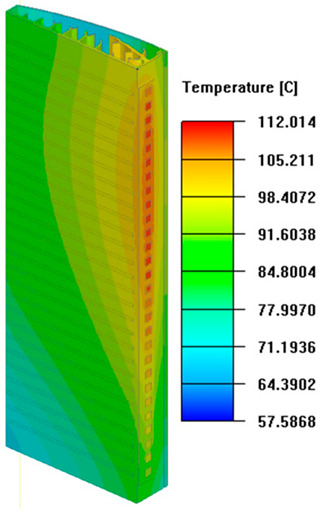	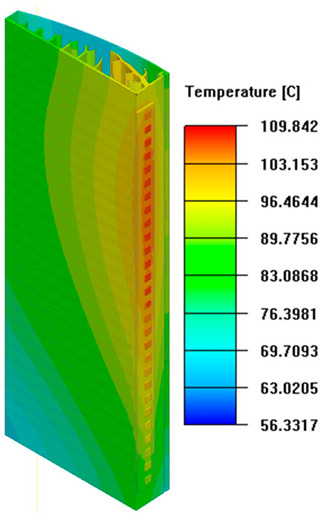
Temperature cloud map in the X-direction	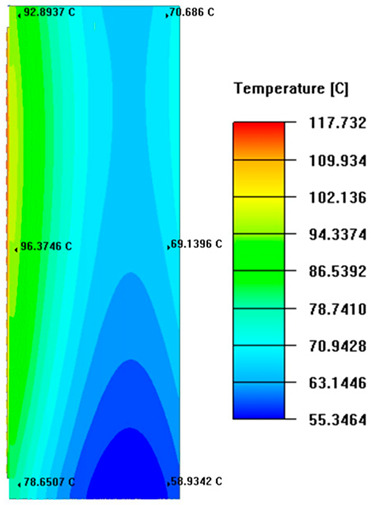	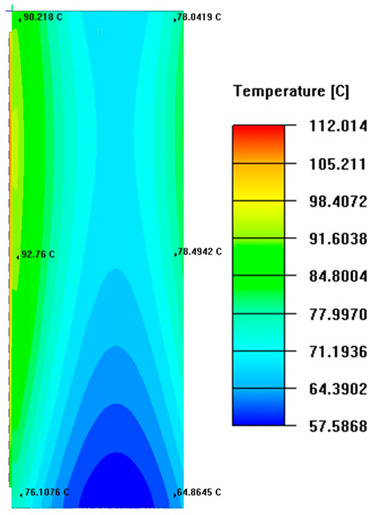	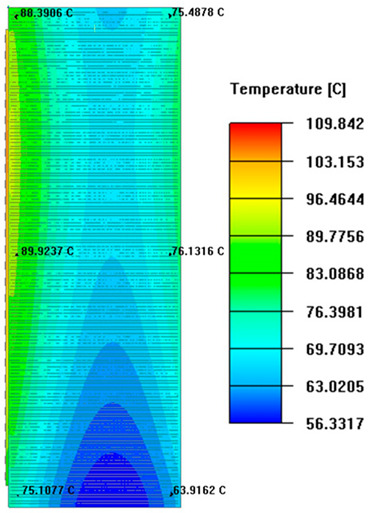
Global air velocity slice cloud map	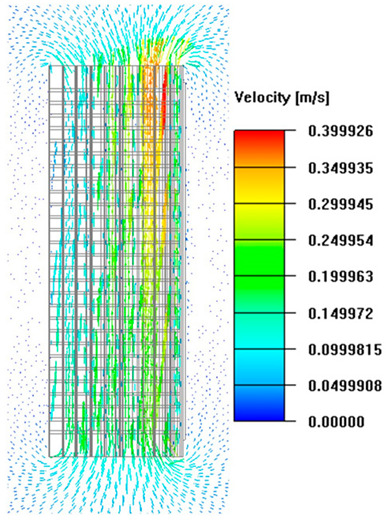	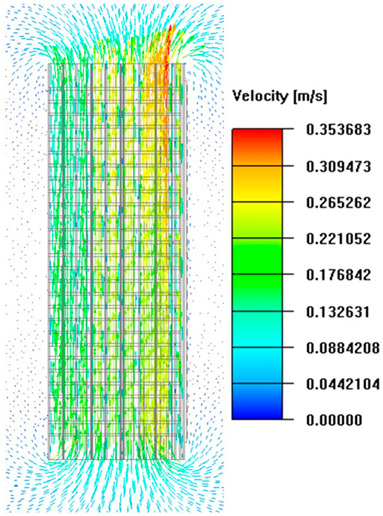	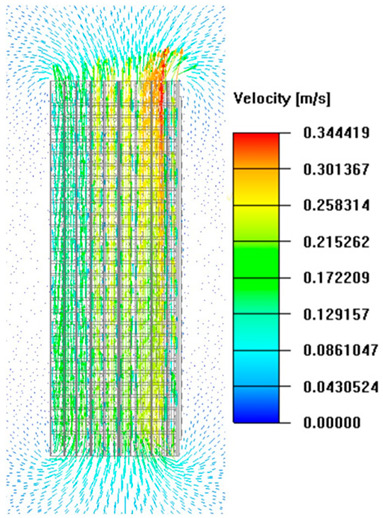

## Data Availability

The data is contained in this paper.
